# Pesticides and brain cancer linked in orchard farmers of Kashmir

**DOI:** 10.4103/0971-5851.76191

**Published:** 2010

**Authors:** Abdul Rashid Bhat, Muhammed Afzal Wani, A. R. Kirmani, T. H. Raina

**Affiliations:** *Department of Neurosurgery, Sher-I-Kashmir Institute of Medical Sciences, Srinagar, Kashmir – 190011, India*

**Keywords:** *Brain cancer*, *Kashmir*, *pesticides*, *orchard farmers*

## Abstract

**Background:**

The atmosphere of valley of Kashmir is ideal for fresh and dry fruit production. Millions of tons of pesticides, insecticides and fungicides (chemicals like chlorpyriphos, mancozeb, captan, dimethoate, phosalone, etc.) are being used by the orchard farmers to spray the plants, fruits and the leaves every year. The increasing trend in the incidence of primary malignant brain tumors in orchard farmers of Kashmir is alarming.

**Aim:**

To determine the relationship between the patients of primary malignant brain tumors and their occupation.

**Materials and Methods:**

Retrospectively case files along with death certificates of 432 patients of primary malignant brain tumors and 457 controls (non-tumor neurologic diseases), admitted for treatment simultaneously over a period of 4 years from January 2005 to December 2008, to the Department of Neurosurgery, Sher-I-Kashmir Institute of Medical Sciences (SKIMS), Kashmir, were studied. Follow-up and family contact was established. The serum cholinesterase activity was measured by kinetic/DGKC calorimetric method and ethylenediaminetetraacetic acid (EDTA) samples were sent to the laboratory. The results are expressed in U/l which is U/l×1000. The laboratory at SKIMS, Srinagar, and Dr Lal PathLabs at New Delhi used a reference range for serum cholinesterase as 3167–6333 U/l.

**Results:**

Analysis revealed that 90.04% (389 out of 432) patients were orchard-farm workers, orchard residents and orchard playing children exposed to the high levels of multiple types of neurotoxic and carcinogenic (chlorpyriphos, dimethoate, mancozeb and captan) chemicals for more than 10–20 years. About 31.9% (124 out of 389) of these from both sexes were younger than 40 years beginning exposure at an early age and had higher (<6334 U/l) serum cholinesterase (SCE) levels. The 9.96% (43 out of 432) patients were not exposed to pesticides. On the other hand, only 119 patients out of 457 controls had recorded history of pesticide exposure and 338 were unrelated to pesticides. Out of 389 patients, 71.7% (279 out of 389) were males and 28.3% (110 out of 389) including 7 members of three families, 6 were females and 1 male.

**Conclusion:**

All orchard-related 389 patients had high grade tumors as compared to the non-pesticide tumors. Mortality in pesticide exposed tumors was 12%. Higher levels of SCE were found in 31.9% (124 out of 389) patients and decreased levels in only 45.3% (176 out of 389) orchard-related patients. The significantcase/control odds ratio (OR) of 0.28, hospital control SCE OR of 1.1 and family control SCE OR of 1.5, points the finger of suspicion toward the link between pesticides and brain cancer.

## INTRODUCTION

Occupational health hazards are well known. The widespread use of pesticides in the agricultural industry to control the insects, pests and fungus and to enhance the crop and fruit production is recognized as a major chemical health hazard for the orchard workers, residents and children by the direct contact and by polluting the aerial, soil and water environment. The residual concentrations of these toxic chemicals in the farm workers lead to a variety of neurological dysfunctions.[1–3] Because of the similarity in the brain biochemistry, the pesticides are particularly neurotoxic to the humans and the most lethal are organophosphates, carbamates and ethylenebisdithiocarbamates (EBDC).[4] The primary action of the organophosphates and carbamates is to irreversibly inhibit the activity of the enzyme acetylcholinesterase (AChE) that hydrolyzes the neurotransmitter acetylcholine in both the peripheral and central nervous systems. This causes accumulation of acetylcholine at cholinergic synapses, leading to overstimulation of muscarinic and nicotinic receptors and thus neurotoxicity.[5] A variety of occupational exposure of workers in industries like rubber, oil refinery, chemical plant and polyvinyl chloride have been reported to have the elevated risk of developing brain tumors.[6] The etiologic importance of exposure to pesticides has been reported by case control studies on childhood brain tumors arising after exposure to the chlordane and heptachlor.[7,8] This was confirmed by a report on patients who died from malignant tumors among whom a high level of organochlorine compounds was found in the adipose tissue of those who had glioblastomas.[9] A study reported two of seven patients from a cluster of primary brain tumors who were exposed to the pesticides.[10] Pesticides are suspected to be the potent risk factors for the lethal brain tumors, especially gliomas, in the children and adults.[11] Annual European Union (EU) pesticide use includes 0.108 million tons of fungicides, 0.08 million tons of herbicides, 21,000 tons of insecticides and 7000 tons of growth regulators – amounting roughly to half a kilogram of active substances for every man, woman and child living within the European Union (EU).[12] The fruit production of Kashmir province of Jammu and Kashmir (J and K), India, is 1.5 million metric tons annually from a total orchard area of 0.2 million hectares which is sprayed and fogged with 7750 MT of fungicides and 3186 MT of insecticides right from March to November every year in 10 recommended scheduled stages from green tip and pink budding of the trees (pre-bloom) up to and after the harvest of the fruits (post-harvest), though unofficial frequency of unscheduled sprays by farmers is increased to 15–20. The excessive use of synthetic pesticides for the last three decades and increase in admission of high grade malignant brain tumors, with history of pesticide exposure, to the neurosurgical center Sher-I-Kashmir Institute of Medical Sciences (SKIMS), Kashmir, in the last 10 years has elicited high degree of suspicion of a link between the pesticides and malignant brain tumors (brain cancer).

## MATERIALS AND METHODS

The Department of Neurosurgery, SKIMS, Srinagar, Kashmir province of J and K, India, caters to about 7–8 million ethnic non-migratory population of Kashmir province as a single center. Since, Kashmir valley has a high potential to produce dry and fresh fruits, pesticides have been used in huge quantities over the last 30 years. All patients with the history of contact with the pesticides are subjected to the serial measurements of serum cholinesterase (SCE) as a protocol. The case files, including death summaries, of 432 patients admitted from January 2005 to December 2008 (4 years) and proved histopathologically as primary malignant brain tumors, were studied. The controls, 457 patient files with non-tumor brain conditions like brain abscesses, tuberculomas, meningitis, strokes, multiple sclerosis and epilepsy, admitted during the same period were also studied. All metastatic lesions to brain were excluded. The history [exposure directly (occupational) of farm workers and indirectly of residents and playing children], clinical, biochemical, radiological and histopathological findings were recorded. The patients and the families were contacted to collect further information, follow-up and to select randomly the familial controls. Further 50 controls from the families were included and 50 more controls selected from general population randomly after age, sex and socioeconomic matching, to make a total of 557 controls. The historical information collected were: age, sex, socioeconomic status, type of work exposure, e.g., mixer-loader applicators, sprayer, fogger in case of adults, number and type of chemicals exposed to, lifelong jobs, orchard guards and supervisors, whether following pesticide applicator precautions like avoiding use of expired drugs or spurious drugs, use of hand gloves, hand wash, masks and goggles, head gear and uniform, eating unwashed fruits from the orchards, location of residential house, location of drinking water source, location of playgrounds of children and frequency of female family members visiting and working in the orchards, age at exposure, duration of exposure, age at the onset of symptoms after exposure. The SCE was measured at the time of admission, indoors and before discharge of patient and the control. The mean of the three estimates was calculated and recorded. The data support the use of sequentialpostexposure plasma cholinesterase analyses to confirm the diagnosisof organophosphate-induced illness in the absence of baselinevalues.[13] The SCE activity was measured by kinetic/DGKC (German Society of Clinical Chemistry) calorimetric method and ethylenediaminetetraacetic acid (EDTA) samples were sent to the laboratory. The results are expressed in kU/l which is U/l×1000. The laboratory at SKIMS, Srinagar, and Dr. Lal PathLabs at New Delhi used a reference range for SCE as 3167–6333 U/l. Two major forms of cholinesterase exist in vertebrates which hydrolyze acetylcholine. The plasma cholinesterase (pseudo- or butyryl-cholinesterase) is found in plasma, liver, pancreas and intestinal mucosa (liver being the main organ). Variations occur due to liver disease, chronic inflammation, malnutrition, morphine, codeine, succinylcholine administration and hypersensitivity reactions. The RBC cholinesterase (true, specific cholinesterase) is found in nervous tissue, erythrocytes, lung, spleen and gray matter. It is decreased in pernicious anemia and after anti-malarial therapy. The estimation of AChE level in circulation is theoretically preferred in organophosphorus poisoning since it would reflect the degree of inhibition of synaptic cholinesterase at motor end plates. But, in practice, estimation of SCE has an advantage because the measurement is simpler and more accurate than the estimation of AChE.[13–16] The SCE levels can indicate the prior presence of cholinesterase inhibition even after recovery of AChE activity by pralidoxime in organophosphorus poisoning,[17] though in acute poisoning of organophosphates, the confirmation of diagnosis depends on demonstrating reduced cholinesterase activity in the circulating blood and the activity is expressed as percentage of normality of healthy adults.[13] But in Kashmir study (with increased or high normal SCE), the organophosphates may have compound specific effects (non-cholinergic) unrelated to the common AChE inhibition, as shown by the similar effects of two organophosphates like chlorpyriphos and diazinon on the gene expression of neonatal rat brain with the doses not inducing biologically significant AChE inhibition and yet both have notable disparities. The disadvantages of SCE estimations are that the normal values are widely variable from one person to another as well as in the same individual at different times and low levels have been observed in some disease states and may also be genetically determined. The serum AChE levels of patients and hospitalized controls were recorded three times and the mean level was considered, and similarly, the levels were checked in the family and general controls. The patients with history of exposure to radiation and anti-mitotic drugs for whatever disease were excluded. The patients and controls with carcinomas, metastasis, hepatitis, acute infection, cirrhosis, nephrotic syndrome, thyrotoxicosis, hemochromatosis, muscular dystrophies and psychiatric disorders were excluded. The data collected were compiled, and the SPSS version 11.5 statistical program was used to compute odds ratio (OR) adjusted for the matching variable (age, sex, orchard workers, non-pesticide exposed cases, SCE). The law of variance was applied wherever required.

## RESULTS

### Background

The atmosphere of valley of Kashmir is ideal for fresh and dry fruit production, which is the major economic source of the Kashmiris [Table 1]. The fruit production area spreads over around 0.2 million hectares of land, of which 0.11 million hectares (>50%) are under apple production, involving about 40% population of the Kashmir directly as orchard farmers, chemical sprayers, etc., and indirectly like children playing in and around orchards, residential houses in orchards, etc. Millions of tons of pesticides, insecticides and fungicides (chemicals like chlorpyriphos, mancozeb, captan, dimethoate, phosalone, etc.) are being used by the orchard farmers to spray the plants, fruits and the leaves at different stages of growth to avoid the infestations and destruction of the fruits. For the last three decades, the farmers have favored and adapted to the newer synthetic but hazardous fungicides and pesticides, never applied before, to enhance the fruit production by replacing the older relatively non-hazardous inorganic sulfur [Table 1]. The incidence of the malignant brain tumors in Kashmir has shown an upward surge in the last 10 years, especially in the orchard farming districts.

**Table 1:** Kashmir orchard area, number of orchard-farm patients, type of pesticide usage and approximate consumption

Orchard district	Orchard area (ha)	Pesticides used (MT)			No. of cases
		Chlorpyriphos	Mancozeb	Captan	
Budgam	29,572	Pink-bud stage 50%, 3 l/ha	Fruitlet stage 30%, 12 kg/ha	Fruitlet stage 10%, 12 kg/ha	55
Anantnag	28,697	Fruitlet stage 50%, 4 l/ha	Pre-harvest stage 30%, 12 kg/ha	Pre-harvest stage 20%, 12 kg/ha	63
Barmaulla (Varmul)	28,031				88
Kupawara	25,583				45
Shopian	24,073				50
Kulgam	18,926				34
Pulwama	17,664				25
Others (Srinagar, etc)	20,563				29
Total	193,109	3186 MT	3400 MT	4350 MT	389

ha = Hectares; MT = Metric tonnes; lit = Litre; kg =Kilogram; Chlorpyriphos = Mancozeb and captan are EU labeled carcinogens

### Age and sex

**Figure 1:**
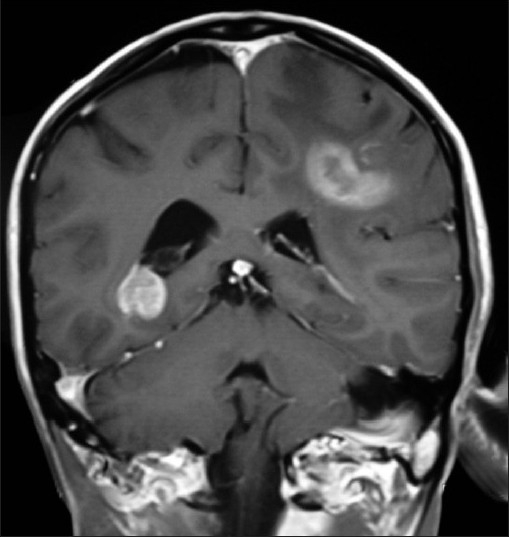
T1WI coronal MRI brain of an orchard worker showing multicentric glioma

**Figure 2:**
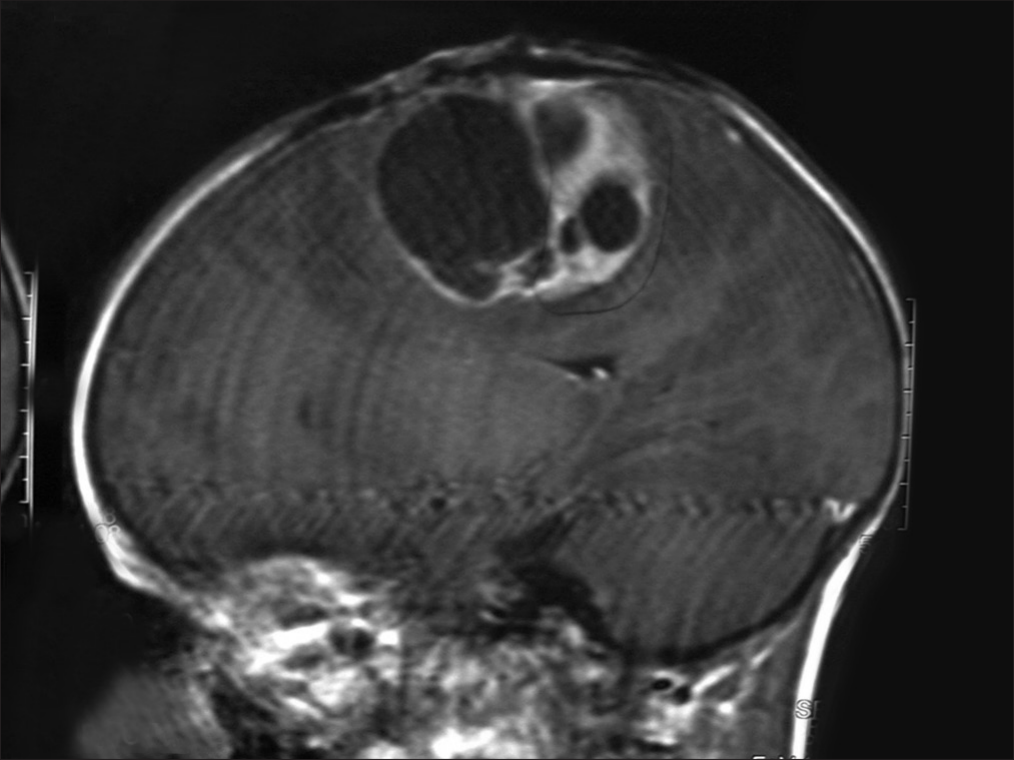
Saggital MRI brain with a glioblastoma multiforme in a male farm worker

The 389 (90%) cases out of 432 primary malignant brain tumors (excluding metastatic lesions), proved by histopathology after open or closed biopsy, were orchard-farm workers in various ways, while 43 had no pesticide exposure [Figures 1 and 2]. Among the 457 controls, only 119 had pesticide exposure. Out of 389 (100%) patients, there were 31 (7.9%) children, 304 (78.1%) adults and 54 (13.9%) elderly people [Table 2]. A total of 279 (71.7%) males and 110 (28.3%) female patients were exposed to pesticides, including 6 females and one male from three orchard residential families. The eldest patient was a 75-year-old male with a hemispheric glioblastoma multiforme and the youngest was a female infant with medulloblastoma. A mortality of 12% (47 cases out of 389) was revealed among orchard (pesticide exposed) farm workers as compared to 7% (3 out of 43) deaths in non-pesticide workers.

**Table 2:** Exposure data related to age and sex

Age and exposure	Males	Females	Total
Age			
Birth to 18 years	70	35	105
19–40 years	166	62	228
41–60 years	43	13	56
61–80 years	0	0	0
Total	279	110	389
Duration of exposure before the onset of symptoms			
Up to 5 years	34	9	43
5–10 years	78	17	95
10–20 years	122	51	173
20–30 years and more	45	33	78
Total	279	110	389
Age at onset of symptoms			
Birth to 10 years	7	4	11
11–20 years	29	11	40
21–40 years	87	37	124
41–60 years	118	42	160
61–80 years	38	16	54
Total	279	110	389

Patients of age from that of an infant to 75 year old were involved; 81 orchard resident families had 85 patients of brain cancer; Familial brain cancer was found in three residential families: One family with brain tumors in mother and daughter, other family had tumors in three daughters; and third family had two siblings (sister and brother). 23 pregnant females and 11 lactating mothers and 16 pregnant woman delivered babies with CNS congenital malformations

### Mode of exposure

(a) Direct, 61.18% (238 out of 389) orchard-farm patients

- Pesticide mixers, sprayers, foggers and orchard tillers
- Females as residents or frequent visitors to help men, collect wood and as part-time orchard helpers
- Men and women as weed handlers, fillers and orchard supervisors
- Avoiding use of hand gloves, hand wash, masks and goggles, head gear and uniform, while using short applicators

(b) Indirect, 38.81% (151 out of 389) orchard-farm patients

- Children either lived in the residential houses with their parents or had exposure by spending most of the time schooling and playing in and around orchards
- The drinking water from the pesticide contaminated orchard wells was source of pesticide exposure for all ages and sexes
- Residential houses constructed in the orchard areas are at risk of pesticide contamination due to unwashed footwear of farm workers, storing farming tools, usage of orchard eatables like vegetables and fruits, hay stacks from orchards carried into homes to store for cattle and contaminated orchard dust carried by the winds into orchard homes

### Age at exposure

The 58.61% (228 out of 389) orchard-farm patients had pesticide exposure at an age of 19–40 years, including 23 pregnant women and 11 lactating mothers, and most (72.80%, 166 out of 228) of these were males, while 26.99% (105 out of 389) patients, mostly children, had pesticide exposure at andbelow 18 years [Table 2]. There was no history of exposure beyond 61 years of age in any orchard-farmpatient.

### Duration of exposure before onset of symptoms

Most of the orchard-farm patients, i.e., 44.47% (173 out of 389) had spent 10–20 years of life exposed to pesticides directly and/or indirectly and 70.52% (122 out of 173) were males. Also, 31.9% (124 out of 389) of these from both sexes were younger than 40 years, initiating exposure at an early age and had higher (<6334 u/l) SCE levels, while 20.05% (78 out of 389) orchard-farm patients had 20–30 years of history of pesticide exposure [Table 2]. A group of 9.16% (54 out of 389) elderly (61–80 years) orchard-farm patients, mostly weed handlers, fillers and orchard supervisors, with high grade brain cancer had lifelong pesticide exposure.

### Age at onset of symptoms

About 73.00% (284 out of 389) orchard-farm patients presented with symptoms and signs related to brain cancer at the age of 21–60 years and most of these (56.33%, 160 out of 284) were between 41 and 60 years of life. Most of the males (73.47%, 205 out of 279) presented in the age group of 21–60 years of life, while 35.48% (11 out of 31) children presented with the malignant brain lesions at the age of 10 years and below [Table 2]. Only about 13.88% (54 out of 389) orchard-farm patients, mostly males (70.37%, 38 out of 54), presented in the elderly age group of 61–80 years. The all elderly age group orchard-farm patients had high grade brain cancer. There were 81 orchard residential families, 85 members of them suffered malignant brain tumors with 6 female (2 adults and 4 children) and 1 male (child) members from three families, i.e., mother/daughter duo from one and three sisters from another family and brother/sister from third family. The 31 children either lived in the residential houses with their parents or had exposure by spending most of the time schooling and playing in and around orchards.

### Pesticide abuse among orchard farmers

**Figure 3:**
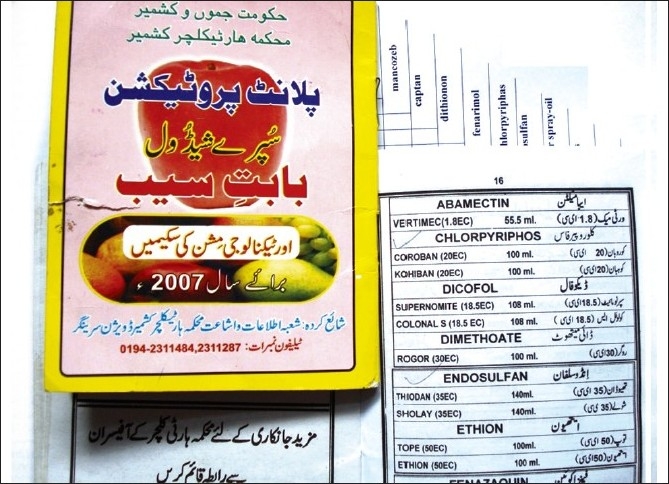
Violation of officially recommended spray schedules in the orchard farms of Kashmir is rampant

**Figure 4:**
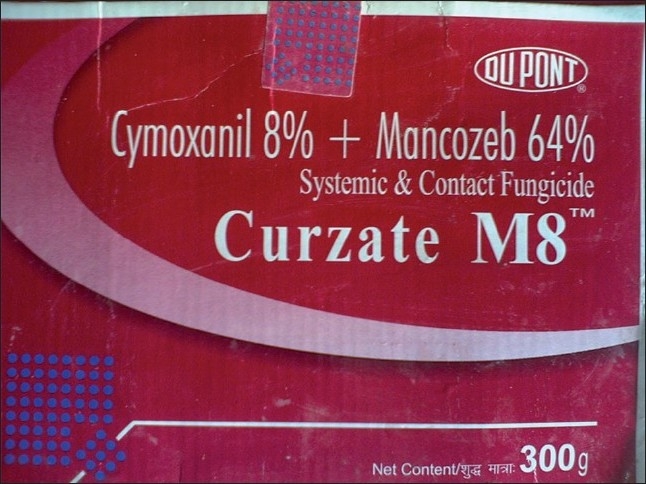
The fungicide, mancozeb (EBDC), a carcinogen, has been long in use in all orchards of Kashmir

**Figure 5:**
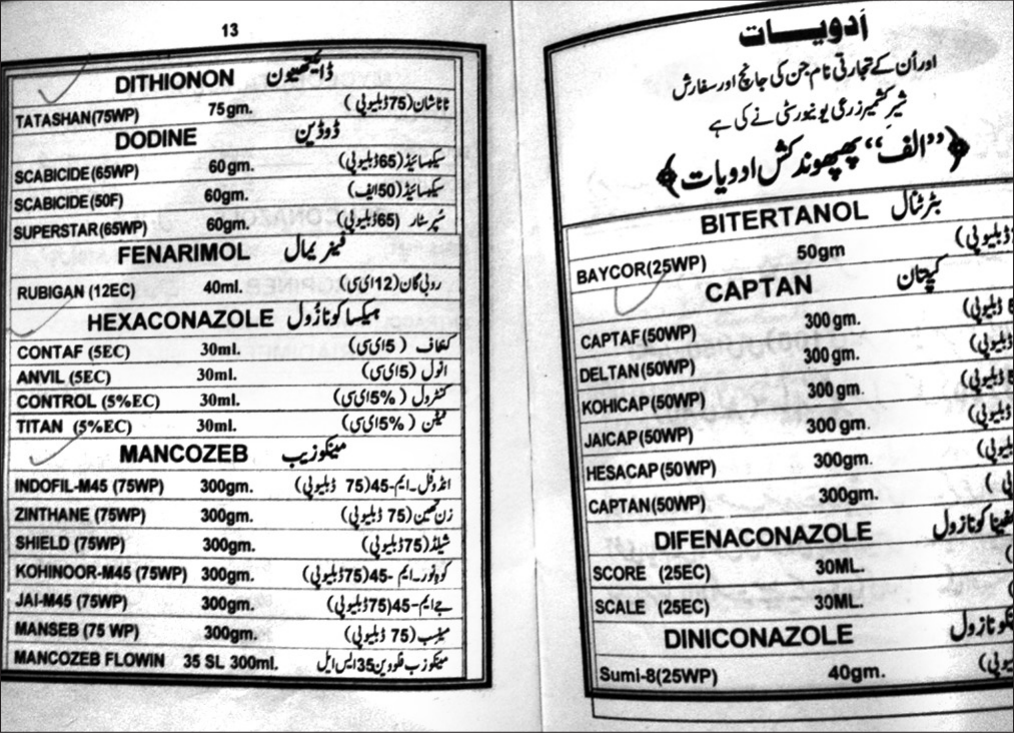
All types of pesticides, irrespective of their health hazardous activity, are used by the Kashmiri farmers

The orchard area with most cases (88 patients out of 389 cases) was Sopore, Baramulla (Varmul) and 63 cases were from Anantnag, followed by the Budgam, Shopian, Kupwara, Srinagar, etc. The orchard area of these districts amounts to about 140,000 ha of a total of 0.193 million hectares, with an annual consumption of thousands of metric tonnes of pesticides when calculated at the officially recommended doses. The officially recommended dithiocarbamates fungicide (EU labeled carcinogen mancozeb) has been sprayed over in the apple orchards at a dose of 12.00 kg/ha twice in a season (Plant Protection Spray Schedule, Information Office, Department of Horticulture Kashmir Division, Srinagar) [Figure 3] in fruitlet stage (pen size) and pre-harvest stage, which alone amounts to about 700 MT (metric tonnes) per season [Table 1]. Mancozeb is in use for the last 30 years in the Kashmir valley Figure 4. Meanwhile, this fungicide is much abused unofficially by the farmers, by its excessive use on the apple trees in the stages not recommended and on fruits like walnut, almond, cherry, etc not recommended. [Figures 3 and 5]. Similarly, the use of captan, a dicarboximide fungicide, EU labeled carcinogen, used excessively by the orchard farmers than the recommended doses of 12.00 kg/ha, is directly absorbed through skin, inhalation and ingestion. This was extensively sprayed in all stages and seasons of fruit growth by the farmers without wearing any special body gear or uniform, and bare handed with short applicators [Figure 5]. Among organophosphates, the most used chemicals were chlorpyriphos (dose 50%, 4 l/ha) and dimethoate [Figure 3], both are neurotoxic insecticides and depress the serum AChE levels [Tables 1 and 3]. Many of these are carcinogens, e.g., chlorpyriphos, captan, mancozeb, etc [Table 1]. The 207 males out of 304 adults (age 19–50 years) were mostly pesticide mixers, sprayers, foggers and orchard tillers using short applicators with bare hands, naked eyes, without any body gear and airway protection. The 97 adult females were frequent visitors and part-time orchard workers. Of these, 23 pregnant females had been exposed to the pesticides in their antenatal and postnatal periods and 11 were lactating mothers. Most adults (44.47%, 173 out of 389 orchard-farm patients) had more than 10–20 years working history in different (apple, walnut, almond, cherry, pear, grapes, peach, apricot, etc.) orchards [Table 2]. Among organochlorines, endosulfan has been the choice of farmers, which is a known convulsant, mutant and carcinogen. This is used on all trees in almost every stage of fruit growth [Figure 3]. The farmers had used cheaper and spurious drugs in greater quantities, than quality and officially sampled drugs. The instructions labeled on the pesticide packs, including date of expiry, were neither attended to nor followed by the farmers. Drinking water from the pesticide contaminated orchard wells was source of pesticide exposure for all ages and sexes. Out of 432 cases, 43 (non-pesticide) primary malignant brain tumors were not associated in any way with orchards or pesticides. From 457 hospital controls, only 119 controls had history of pesticide exposure and 338 had no relation to pesticides (OR=0.28, significant). Residential houses constructed in the orchards are at risk of pesticide contamination due to unwashed footwear of farm workers, farming tools, vegetables, fruits, hay stacks for cattle carried into homes and contaminated orchard dust carried by the winds. The drinking water wells in the orchards and orchard residential houses are contaminated due to the direct spill of the chemical mixture into the wells while constituting the spray and indirectly by the contaminated soil washings drained by rain into the drinking wells. Thus, drinking water site is the persistent source of pesticide exposure.

**Table 3:** Serum cholinesterase (SCE levels in orchard-farm worker patients and non-pesticide exposed patients and controls

Cases/controls	SCE levels (U/l)			Total
	Decreased (<3167)	Normal (3167–6333)	Increased (>6333)	
Cases				
Orchard-farm workers	176	89	124	389
Non-pesticide	1	38	4	43
Subtotal	177	127	128	432
Hospital controls				
Orchard-farm workers	2	10	7	19
Non-pesticide	17	354	67	438
Subtotal	19	364	74	457
Family controls				
Orchard-farm workers	6	20	8	34
Non-pesticide	4	4	8	16
Subtotal	10	24	16	50
General control				
Orchard-farm workers	2	6	1	9
Non-pesticide	5	19	17	41
Subtotal	7	25	18	50
Grand Total	213	540	236	989

*P* value=0.0001; Hospital controls SCE: OR=1.1; Family controls=1.5; Case/control: OR=0.28; Depressed SCE levels in 82.6% (176 orchard farmers out of 213 controls/ cases, Whether orchard farmers or non-pesticide exposed) patients predicts even more frequency in orchard-farm workers, though variations in Kashmiris are common

### Symptoms and signs

**Figure 6:**
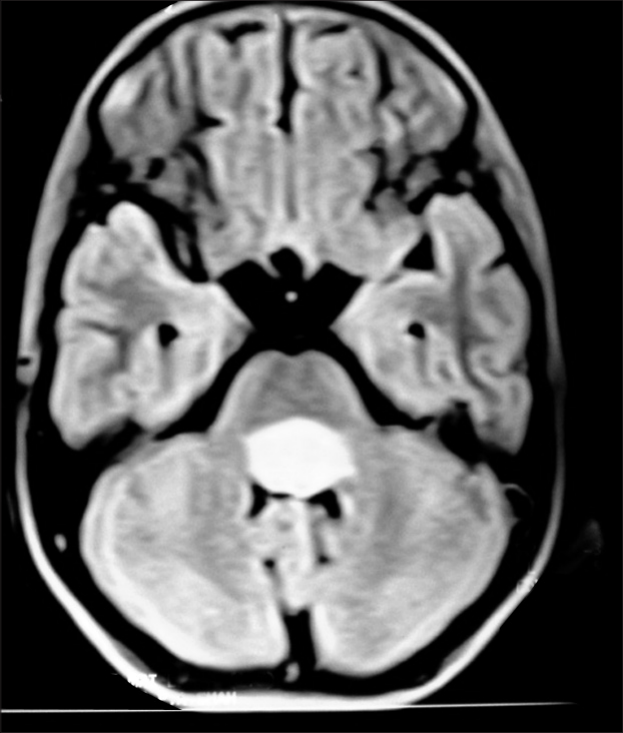
Fourth ventricular ependymoma on T1W axial MRI brain of a 10 year old brother, whose 13 year old sister Figure 7 has right diencphalic oligodendroglioma

**Figure 7:**
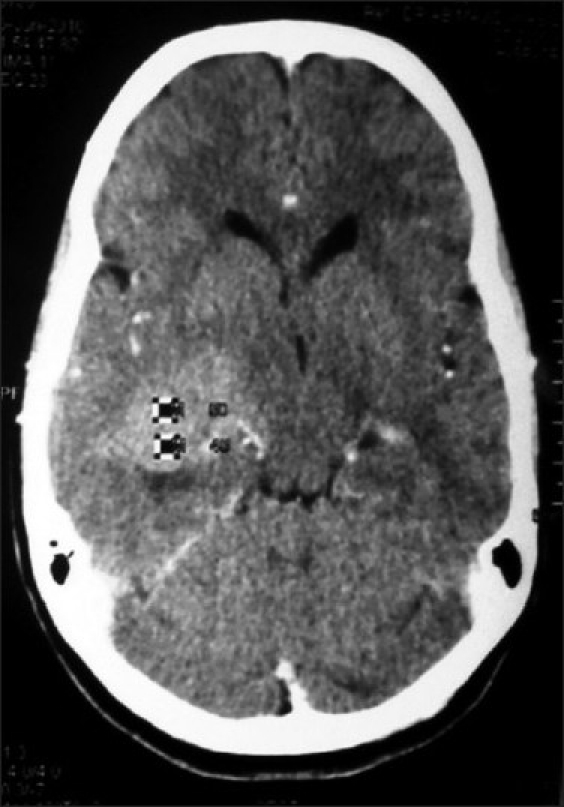
Right diencephalic oligodendroglioma on contrast CT-scan brain in a 13 year old sister, whose 10 year brother (Figure 6) has fourth ventricular ependymoma

The most common presenting symptom was headache, followed by the epilepsy, vomiting and the visual blurring. The most common sign found was papilloedema. Computed tomography (CT) scan and magnetic resonance imaging (MRI) brain were the diagnostic tools of choice [Figures 1, 2, 6 and 7]. All patients were operated upon and histological diagnosis sought and recorded.

### Incidence and histological types of malignant brain tumors

From January 2005 to December 2008, each passing year showed an increase in the incidence of new cases of highly malignant brain tumors like glioblastoma multiforme and medulloblastomas; most, i.e., 29.30% (114 out of 389) patients reported in 2008 as compared to 20.82% (81 out of 389) patients in 2005. Such a trend is not seen in case of non-pesticide tumors [Table 4]. Most of the 389 orchard-farm workers with malignant brain tumors were having highly malignant astrocytomas, glioblastoma multiforme, anaplastic oligodendrogliomas, ependymomas, choroid plexus papillomas, medulloblastoma, etc., as compared to the 43 non-pesticide malignant brain tumors. The children had mostly primitive neuroectodermal tumors. Some of the patients had high grade multicentric type of gliomas with worst prognosis [Figures 1 and 2]. The elderly age group (61–80 years) of 54 orchard-farm workers had all worst grade (WHO grade IV) of brain cancer (glioblastoma multiforme).

**Table 4:** Histological types of primary malignant brain tumors and incidence in orchard-farm workers and non-pesticide patients from 2005 to 2008

Histological type	No. of orchard worker patients/year					No. of nonpesticides patients
	Total	Years				
	4 years	2005	2006	2007	2008	
Glioblastoma multiforme (WHO grade IV)	96	16	18	26	36	5
Anaplastic astrocytomas (WHO grade III)	67	13	15	18	21	4
Astrocytoma (WHO grade II)	38	8	7	12	11	15
Anaplastic oligodendroglioma (WHO grade III)	28	6	8	7	7	2
Oligodendroglioma (WHO grade II)	30	9	8	9	4	11
Anaplastic ependymoma (WHO grade III)	21	4	6	7	4	—
Ependymoma (WHO grade II)	28	7	7	6	8	6
Anaplastic oligo-astrocytoma (WHO grade III)	7	3	1	2	1	—
Mixed oligo-astrocytoma (WHO grade II)	10	4	2	1	3	—
Gliosarcoma	11	4	4	3	—	—
Gliomatosis cerebri	6	—	1	2	3	—
Choroid plexus papilloma	19	5	5	4	5	—
Ganglioglioma	5	—	1	2	2	—
Esthesio neuroblastoma	2	—	—	1	1	—
Pineocytoma	3	—	1	1	1	—
Medulloblastoma	15	2	4	4	5	—
Retinoblastoma	3	—	—	1	2	—
Total	389	81	88	106	114	43

Some patients had high grade multicentric gliomas; Mortality in cases: 12% in orchard workers and 7% in non-pesticide tumors; Glioblastoma multiforme and medulloblastomas show increase in the incidence from the year 2005 to 2008

### Pesticides and cholinesterase

The number of pesticides used by the orchard farmers in Kashmir was more than 30 fungicides, insecticides, acaricides, etc., with most of these spurious. Among these, chemical groups like organophosphates, organochlorines, carbamates, EBDC, pyrethroids, phosphines, dicarboximides, inorganics, ureas, dinitroanilines, etc., are used at large scales. The officially recommended dithiocarbamate fungicide (EU labeled carcinogen mancozeb) has been used in the apple orchards at a dose of 12.00 kg/ha twice in a season [Figure 5] in fruitlet stage (pen size) and pre-harvest stage, which alone amounts to about 700 MT per season [Table 1]. Mancozeb is in use for the last 30 years in the Kashmir valley [Figure 4]. Similarly, captan, a dicarboximide fungicide, EU labeled carcinogen, used excessively by the orchard farmers than the recommended doses of 12.00 kg/ha, is directly absorbed through skin, inhalation and ingestion. Among organophosphates, the most used chemicals were chlorpyriphos (dose 50%, 4 l/ha) and dimethoate. Both are neurotoxic insecticides and depress the serum AChE levels [Tables 1 and 3]. However, results revealed that only 45.3% (176 out of 389) patients of those exposed to pesticides for 5–10 years had lower SCE levels of <3167 U/l. This also revealed normal SCE (3167–6333 U/l) in 22.8% (89 out of 389) patients and higher levels of >6334 U/l in 31.9% (124 out of 389) patients, equally in both the sexes Table 3. The 31.9% orchard-farm workers with higher (>6334 U/l) levels of SCE were below 40 years and had pesticide exposure of 10–20 years from an early age. But the significantcase/control OR of 0.28, hospital control SCE OR of 1.1 and family control SCE OR of 1.5 point the finger of suspicion toward the link between pesticides and brain cancer [Table 5]. The reason for the altered levels of SCE enzyme may be either different, non-cholinergic, mechanism of action of organophosphates triggered through cAMP or continuous chronic poisoning rather than acute which depresses the AChE levels or a mixture of different pesticides with different actions on the central nervous system. The causes may also be racial, genetic or immunological.

**Table 5:** Retrospective case–control studies which evaluated the pesticide–brain tumor link

Study	No. and source of cases	No. and source of controls	Type of exposure	Method	Results
Thomas *et al*.,1986	718 brain tumor deaths	738 controls	Occupation	Death certificates	OR=0.8; 95%CI=0.4–1.8 (NO)
Speers *et al*.,1988	202 Texas males died of gliomas	238 males	Occupation	Death certificates	OR=0.61; 95%CI=0.3–1.22 (NO)
Musicco *etal*.,1988	420 patients of gliomas hospitalized	465 non-glioma brain tumors and 277 nontumor patients of neurologic disorders	Occupation and residence	Interview	RR=1.6; 9 5%CI=1.06–2.42 (SIG)
Reif *et al*.,1989	452 registered brain cancer patients	19452 non-brain cancer patients	Occupation	Interview	OR=1.3; 95%CI=1.0–1.7 (SIG)
Schlehofer *et al*., 1990	226 patients with primary brain tumors in Germany	418 population controls	Occupation	Questionnaire	RR=1.1; 95%CI=0.7–1.9 (NO)
Forastiere *et al*., 1993	1674 male cancer deaths from Italian agricultural region	Random samples of 480 individuals selected from same regional mortality file as being deceased from all causes	Occupation	Regional mortality file (death certificates)	OR=1.04; 95%CI=0.43–2.44 (NO)
Rashid *et al*. (Kashmir study)	Out of 432 patients of malignant brain tumor hospitalized, 389 orchard-farm workers	Of 457 hospital controls, 119 orchard farmers, 50 family and 50 general controls	Occupation and residence	Hospital files, medical records and family/patient interaction	Case/control: OR=0.28; hospital control SCE: OR=1.1; family control SCE=1.5

### Familial gliomas

Three families among 81 orchard residential families had more than one member having primary malignant brain tumor. The first family belonged to Baramulla (Varmul) and the two members were mother with anaplastic astrocytoma and her 10 year-old daughter with a medulloblastoma. The second family comprised three sisters from the Srinagar district, who presented from eldest to youngest in a time span of 3 years. The eldest sister had ependymoma, the younger one had medulloblastoma and the youngest had choroid plexus papilloma. Third family had two siblings, one 13 year sister with diencphalic oligodendroglioma and other 10 year brother with fourth ventricular ependymoma. The reason for familial primary malignant brain tumors could be common stimulatory agent. These three families revealed extensive use of multiple pesticides including mancozeb, chlorpyriphos and captan. The history revealed that about 16 female patients of primary brain cancer had multiple abortions, still births and babies delivered with congenital anomalies of brain and spinal cord.

## DISCUSSION

The present study on the orchard farmers of Kashmir shows an every year incidental increase in high grade brain cancer (glioblastoma multiforme and medulloblastomas) from January 2005 to December 2008. This study also revealed 31 children with brain cancer, the youngest being an infant. The prenatal, natal and postnatal exposure to pesticides has created enough of doubt concerning the toxicity and mitotic abnormalities in the developing brain of pregnant patients. The 16 female patients had delivered babies with congenital central nervous system malformations. Endosulfan has been one of the most used and abused pesticides. Chlorpyriphos is the most extensively studied organophosphate, with respect to the neurotoxicity, in the laboratory models, where prenatal and neonatal exposure has lead to a variety of behavioural abnormalities in both the mice and rats. Chlorpyriphos exposure in rat embryo cultures at concentrations comparable to those found in human meconium showed mitotic abnormalities and apoptosis during the neural tube development stage. However, exposure during gestation led to deficits in brain cell numbers, neuritic projections and synaptic communication, which emerged in adolescence and continued into adulthood. The deficits elicited by prenatal exposure to chlorpyriphos are evident even at exposures below the threshold for detectable AChE inhibition, i.e., far below the 70% inhibition of AChE required for systemic toxicity in adults. These findings suggest that chlorpyriphos also acts via non-AChE inhibition mechanisms to cause neurotoxicity.[18–21] The Kashmir study shows difference in the levels of SCE among the patients who were pesticide exposed farm workers [Table 3]. The 31.9% (124 out of 389) orchard-farm workers with higher {>6334 U/l} levels of SCE were below 40 years and had pesticide exposure of 10–20 years from an early age The non-cholinergic mechanisms of chlorpyriphos are not clear but a possible target may be the signaling cascades involved in neuronal and hormonal inputs, including the cyclic AMP (cAMP)–protein kinase A cascade, receptor signaling through protein kinase C, and direct effects on the expression and function of nuclear transcription factors mediating the switch from proliferation to differentiation, including c-fos, p53, AP-1, SP 1 and CREB (Ca^2+^ /cAMP response element binding protein).[22] This has opened the possibility that organophosphates may have compound specific effects unrelated to the common AChE inhibition, as shown by the similar effects of two organophosphates, chlorpyriphos and diazinon, on the gene expression of neonatal rat brain with the doses not inducing biologically significant AChE inhibition and yet both have notable disparities.[23–25] The pesticide-exposed orchard farmers of Kashmir with primary brain cancer showed a lot of variation in the levels of SCE. Although 45.3% orchard-farm patients had depressed levels of SCE, 54.7% had normal and higher serum SCE levels with a significantcase/control: OR=0.28; hospital control SCE: OR=1.1; family control SCE: OR=1.5, predicting decrease in SCE levels more often [Tables 3 and 5]. The non-cholinergic mechanism, slow and chronic poisoning with chlorpyriphos and mixed exposure to pesticides may be the probable causes for the neurotoxicity and stimulation of brain cancers in Kashmir. The primary malignant brain tumors in Kashmir are, reportedly, on a rise since 1999, especially among the elderly population. A study showed glioblastomas multiforme accounting for 69.4% of all gliomas.[26] Headache and epilepsy have been the most common symptoms and signs as reported in a study.[27] This is similar to this hospital-based case–control study. Epidemiological studies show associations with neurodevelopmental deficits on exposure to the mixed pesticides. Laboratory experimental studies using model compounds suggest that many pesticides currently used in Europe, including organophosphates, carbamates, pyrethroids, EBDC and chlorophenoxy herbicides, can cause neurodevelopmental toxicity.[4] The organochlorine pesticide, endosulfan, is reported to be lipophilc in nature and neurotoxic.[28] Endosulfan is an epileptogenic and experimentally a teratogenic, tumorogenic, carcinogenic and the human mutation is reported.[29] The manufacturer of chlorpyriphos, DowElanco, Indianapolis Indiana, USA has agreed to restrict its recommended uses in fleas, ticks and pets.[30] A study that assessed mortality rates among vineyard workers in 89 geographic locations in France found a significantly higher incidence of brain cancer among those exposed to pesticides compared to the French population.[31] Similar to this study, the Kashmir study reveals the highest incidence of primary brain cancer in the geographic areas of Baramulla (Sopore, Varmul), Anantnag, Budgam, Shopian and Kupwara which comprises most of the orchard areas of Kashmir [Table 1]. A total mortality of 12% was recorded in the pesticide exposed orchard farmers as compared to 7% non-pesticide patients. Many farmers using fungicides reported the use of commercial compounds of copper sulfate, some of which contain methylurea, a carcinogen of nervous system in animals.[32] In Europe, the grapes receive 15% of total synthetic (active substance) pesticides applied to major crops. The synthetic fungicides applied to grapes include substances like dithiocarbamates, a family of chemicals in which pesticides like maneb and mancozeb are EU classified carcinogens. Among the hazardous pesticide list commonly found in the food items purchased in EU are proven carcinogens like maneb, procymidone, iprodione and captan. While procymidone has 93% and iprodione 100% transfer rate from grapes to wine, both are proven carcinogens as reported by French Ministry of Agriculture.[33,34] Compared to Europe, the Kashmir province of J and K state in India is 1/20th in area. The amount of pesticides and fungicides sprayed are amounting to the thousands of metric tonnes of mancozeb, captan, chlorpyriphos, dimethoate, etc. Familial gliomas have been reported in many studies but not in pesticide workers. The present study recorded three families with six female and one male members having deadly primary brain cancer and some of the cases even with multicentric high grade gliomas. There are many reports where brothers, parent and children in the families suffered similar types of brain cancer.[35,36] Dithiocarbamates are non-cholinesterase inhibiting and sulfur-containing carbamates which are primarily used as fungicides and herbicides. There are four major classes, of which the EBDC like mancozeb, maneb and zineb are EU labeled carcinogens. Mancozeb is linked to the uncoupling of the mitochondrial electron transport chain which generates reactive oxygen species leading to neuronal toxicity.[37] Owing to the rapid dermal, inhalational and oral absorption of the mancozeb, the un-gloved, un-masked and un protectively clothed Kashmiri orchard workers who spray tonnes of this pesticide are much vulnerable to its toxicity and carcinogenic effects [Table 1]. Epidemiologically, it is difficult to study the risk of a specific pesticide as a cause of brain tumor because the exposure is not limited to one chemical only but a mixture of multiple pesticides in a spray or a fog.[32] A case–control study revealed that among household pesticides, pest-strips have been reported to be the most consistent pesticides related to a variety of childhood cancers including brain cancer.[38] The childhood cancers in the pesticide workers of Kashmir study were 7.9% (31 out of 389) and most of these were primitive neuroectodermal tumors which have worst prognosis and fatal outcome [Table 2]. However, authors in an epidemiological review revealed that great majority of cohort studies of chemical workers employed in the manufacture of pesticides did not indicate an excess of brain cancer mortality. But few cohort studies of pesticide applicators showed elevated relative risk for excess mortality due to brain cancer.[39] The present Kashmir study finds substantial amount of evidence in favor of a relationship between the malignant brain tumors (brain cancer) and pesticide workers in the orchard farms of Kashmir with a significant case/control: OR=0.28; hospital control SCE: OR=1.1; family control SCE: OR=1.5 [Tables 3 and 5]. Evaluation of a series of retrospective case–control studies revealed significant link between occupation and the brain cancer. The studies of Musicco *et al*., in 1988, showed a significant Relative Risk (RR) of 1.6 and 95% confidence interval (CI) of 1.06–2.42 and Reif *et al*., 1989, reported a significant OR of 1.3 and a 95% CI of 1.0–1.7. However, Thomas *et al*., and others depicted non-significant relationship between the two.[32,40–44]

## CONCLUSIONS

The association between the malignant brain tumors and pesticide insult is still a dilemma.

This study provides many evidences to link primary malignant brain tumors in Kashmiri orchard-farm workers with pesticides. The study revealed a case/control OR of 0.28. Although chemically there appear prominent variations in the SCE levels between Kashmiris exposed to pesticides and people from other geographic locations, the SCE levels in hospital controls depicts an OR of 1.1 and SCE in family controls shows an OR of 1.5 [Tables 3 and 5] which predicts decreased levels in 45.3% orchard-farm workers more frequently than all controls and 31.9% orchard-farm patients. The SCE levels were higher than 6334 U/l in 31.9% (124 out of 389) orchard-farm patients younger than 40 years and exposed to pesticides for more than 10–20 years from an early age. This may be racial, genetic or else the non-cholinergic mechanisms of chlorpyriphos where the possible target may be the signaling cascades involved in neuronal and hormonal inputs, including the cAMP–protein kinase A cascade. Clinically, the link between the pesticides and brain cancer appears quite strong and possible but accurate epidemiological studies are yet to document this association. This is in part due to lack of study of action of a single pesticide in an individual case because of exposure to multiple pesticides in one time. However, laboratory and animal studies are in favor of such a link. In the future, studies are needed to accurately localize the link. One more worry has been emergence of familial gliomas in pesticide handlers, orchard-farm workers and orchard-residents.
